# Cryptic Diversity in the Monotypic Neotropical Micromoth Genus *Angelabella* (Lepidoptera: Gracillariidae) in the Peru-Chile Desert

**DOI:** 10.3390/insects11100677

**Published:** 2020-10-06

**Authors:** Marcelo Vargas-Ortiz, Guido Aliaga-Pichihua, Ana Lazo-Rivera, José Cerdeña, Jackie Farfán, Wilson Huanca-Mamani, Héctor A. Vargas

**Affiliations:** 1 Programa de Doctorado en Sistemática y Biodiversidad, Departamento de Zoología, Facultad de Ciencias Naturales y Oceanográficas, Universidad de Concepción, Concepción 4030000, Chile; marcelovargasortiz@gmail.com; 2 Escuela Profesional de Biología, Universidad Nacional de San Agustín de Arequipa, Av. Alcides Carrion s/n, Arequipa 040000, Peru; galiaga@unsa.edu.pe (G.A.-P.); alazor@unsa.edu.pe (A.L.-R.); 3 Museo de Historia Natural, Universidad Nacional de San Agustín de Arequipa, Av. Alcides Carrion s/n, Arequipa 040000, Peru; cerdenajoseal@yahoo.es; 4 Programa de Pós-graduação em Biologia Animal, Departamento de Zoologia, Instituto de Biociências, Universidade Federal do Rio Grande do Sul, Porto Alegre, RS 90000-000, Brazil; jjackie4u@gmail.com; 5 Departamento de Producción Agrícola, Facultad de Ciencias Agronómicas, Universidad de Tarapacá, Arica 1000000, Chile; whuanca@uta.cl; 6 Departamento de Recursos Ambientales, Facultad de Ciencias Agronómicas, Universidad de Tarapacá, Arica 1000000, Chile

**Keywords:** allopatric species, cryptic species, genetic divergence, mitochondrial DNA, *Tecoma fulva fulva* (Cav.) D. Don

## Abstract

**Simple Summary:**

The Neotropical Region harbors a highly diverse and poorly known fauna of leaf miners of the micromoth family Gracillariidae (Lepidoptera). *Angelabella* is a genus of Gracillariidae whose geographic range is restricted to a few valleys of the arid environments of the Peru-Chile desert. Only one species is currently included in this genus. The aims of this study were to explore the geographic range, determine the spatial distribution of mitochondrial lineages, and test lineage conspecificity hypotheses in *Angelabella*. The spatial distribution of genetic diversity indicated four spatial clusters, three of which are north of the previously known geographic range. These groups were defined as different species by four species delimitation methods. These results suggest that *Angelabella* harbors at least four morphologically cryptic species with restricted, not overlapping geographic ranges. This study shows that adequate single locus sequence analysis can be useful to discover surprising biodiversity patterns in underexplored environments, providing the base to plan further studies involving little-known organisms.

**Abstract:**

*Angelabella* (Lepidoptera: Gracillariidae: Oecophyllembiinae) is considered a monotypic Neotropical genus of leaf miner micromoths known only from a few valleys of the arid environments of the Peru-Chile desert, particularly the southernmost part of Peru and northernmost part of Chile (type locality), where natural populations of its primary host plant occur. The geographic distribution of potential host plants provides a scenario for a wider range for this micromoth genus. The aims of this study were to explore the geographic range of *Angelabella*, determine the spatial distribution of mitochondrial lineages, and test lineage conspecificity hypotheses. The spatial distribution of genetic diversity indicated the presence of four spatial clusters, three of which are north of the previously known geographic range. Genetic distances were 0.2–0.8% and 3.6–8.3% (K2P) between haplotypes of the same and different spatial clusters, respectively. Phylogenetic relationships indicated reciprocal monophyly among the four spatial clusters, suggesting that allopatric differentiation processes have governed the recent history of *Angelabella* in these arid environments. These groups were defined as different species by four species delimitation methods, suggesting that *Angelabella* is not a monotypic genus, but harbors at least four morphologically cryptic allopatric species with restricted geographic ranges, including the type species and three candidate species.

## 1. Introduction

**Figure 1:**
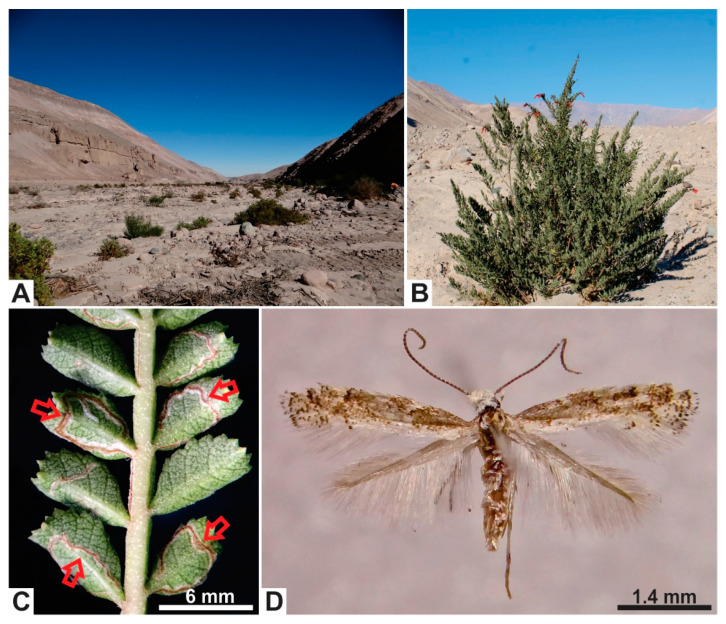
Habitat and host plant of *Angelabella tecomae* (Gracillariidae). (**A**) Habitat of *A. tecomae* in the type locality, the Azapa Valley, northern Chile. (**B**) The main host plant in the type locality, *Tecoma fulva fulva* (Bignoniaceae). (**C**) Detail of a leaf of *T. f. fulva* showing leaflets and winged rachis mined by larvae of *A. tecomae*. (**D**) Female adult of *A. tecomae* in dorsal view.

The Neotropical micromoth genus *Angelabella* Vargas & Parra (Lepidoptera: Gracillariidae: Oecophyllembiinae) includes only the type species, *A. tecomae* Vargas & Parra, a leaf miner described from the Azapa Valley, Atacama Desert of northern Chile, whose primary host plant is the shrub *Tecoma fulva* (Cav.) D. Don (Bignoniaceae) [1] (Figure 1). The small females of *A. tecomae*, forewing length about 3 mm, select actively growing leaflets for egg laying on this shrub [2]. This behavior ensures food availability to complete larval and pupal development inside a single leaflet, generating an aggregated spatial pattern of the immature stages along the shoot with eggs at the apex, larvae at intermediate positions and pupae at the base [2]. Despite the highly specialized egg laying site selection by the females, the host range of *A. tecomae* is not restricted to *T. f. fulva*, as this micromoth is also able to use the exotic ornamental tree *Tecoma stans* (L.) Juss. Ex Kunth as a host plant [3]. *T. stans* is a widespread Neotropical tree mainly associated with the Andes in South America [4]. It is restricted to the urban area in the range of *A. tecomae* [3]. The geographic range of *A. tecomae* appears to be narrow based on the available records, as besides the type locality, this micromoth has been collected only in a second valley in northern Chile and one valley in southern Peru, with about 50 km between the most distant records [3,5].

Taxonomic identification of Lepidoptera is mainly based on adult morphology, with special reference to genitalia structures. However, morphological differentiation can be extremely subtle between adults of different species in some genera of Gracillariidae, especially in those of small size such as *A. tecomae*, hindering identification based exclusively on the morphology of this life stage [6,7]. DNA barcodes [8] are widely recognized as helpful complementary tools in taxonomic identification of Lepidoptera [9,10,11], including Gracillariidae [12,13,14,15]. Their use is especially adequate to identify immature stages [16,17] and to detect putative new species [6,18].

The geographic range of the host plant *Tecoma fulva* extends from the lowlands of the coastal valleys of south-central Peru and northern Chile to the Andes highlands of northern Argentina, Bolivia, and south-central Peru [4]. Six subspecies are currently recognized in this extensive range, four of which are found between near sea level and at about 2800 m elevation on the western slopes of the Andes of south-central Peru [4]: the nominotypical *T. f. fulva*, shared with the coastal valleys of northern Chile, *T. f. arequipensis* (Sprague) J. R. I. Wood., *T. f. tanaeciiflora* (Kränzlin) J. R. I. Wood and *T. f. guarume* (A. DC.) J. R. I. Wood.

As the host range of *A. tecomae* is not restricted to *T. f. fulva*, the presence of additional subspecies of *T. fulva* between the Pacific coast and the Andes of south-central Peru provides potential for a wider geographic range for this micromoth. Accordingly, to verify if *A. tecomae* is more widespread than currently known, it should be surveyed for in habitats where other subspecies of *T. fulva* occur. Considering the patchy distribution of potential host plants between extensive arid areas in the desert landscape of south-central Peru, together with the expected low vagility of this micromoth suggested by its small size, highly specialized egg lying site selection of the females and the endophytic habit of the larvae, the differentiation of mitochondrial lineages is expected to be associated with localities (patches), allowing us to hypothesize that such lineages are reciprocally monophyletic as reported in another micromoth, *Bucculatrix mirnae* Vargas & Moreira (Bucculatricidae), in similar environments [19]. If this hypothesis is validated, we can determine if such levels of differentiation have led to the formation of independent lineages, that is, if they can be considered as species.

Molecular approaches to species delimitation allow testing conspecificity hypotheses through a variety of different methods [20], some based on intraspecific genetic distance thresholds (ABGD [21]), a mixture of speciation models and coalescent theory (GMYC [22,23]) or speciation modeling in terms of number of nucleotide substitutions (PTP, [24]), all allowing single locus delimitation [25,26]. Since a given method makes a number of simplifying assumptions that could be violated in a particular system, it is important to test hypotheses by applying a wide range of species delimitation analyses and to rely on delimitations that are congruent across methods [27].

The aims of this study were to explore the geographic range of *Angelabella*, determine the spatial distribution of mitochondrial lineages and test lineage conspecificity hypotheses using DNA barcodes of individuals collected on potential host plants from the Peru-Chile desert.

## 2. Materials and Methods

### 2.1. Sample Collection

Mined leaflets were collected from individuals of three subspecies of *T. fulva* in four localities of Peru. Identification of the subspecies of *T. fulva* was based on the study of Wood [4]. From north to south, the sampling sites and their respective plants were as follows: (1) Lima Department (n = 4 pupae), Zuñiga, Lunahuana-Yauyos highway (12°50′ S, 75°57′ W), 950 m, June, 2019, *T. f. guarume*; (2) Arequipa Department (n = 5 pupae; n = 2 male adults), Pocsi (16°30′ S, 71°25′ W), 2900 m, August, 2018, *T. f. arequipensis*; (3) Moquegua Department (n = 10 pupae), Yacango (17°05′ S, 70°52′ W), 1900 m, January, 2020, *T. f. fulva*; and (4) Tacna Department (n = 10 pupae), Tacna (18°03′ S, 70°17′ W), 460 m, January, 2020, *T. f. fulva*. The maximum distance between localities was nearly 1000 km. The southernmost locality (Tacna Department) corresponds to the only previous Peruvian record of *A. tecomae* based on morphology [3]. The collected leaflets were dissected under a stereomicroscope to extract pupae, which were placed in ethanol 95% and kept at −20 °C until DNA extraction. Differences in number of individuals collected in each site reflect the availability of samples at the time of field work. The identification of the pupae was undertaken by comparison with pupae from the type locality of *A. tecomae*. In addition, two male adults were obtained from pupae from Arequipa; their genitalia were dissected and compared with males of *A. tecomae* from the type locality.

### 2.2. DNA Extraction and Sequencing

Genomic DNA was extracted from 29 pupae following the procedures described in [28]. A fragment of the COI gene was amplified by polymerase chain reaction (PCR) with the primers LEP-F1 and LEP R1 [29]. PCR reactions were performed in a final volume of 20 μL. Each reaction contained 1 μL of DNA extract, 10 ρmoles of each primer, 2.5 mM of each dNTP, 2 mM MgCl_2_, 1X PCR buffer (KCl), 1 unit of Taq DNA polymerase (Thermo Scientific) and sterile distilled water. The amplification program was 5 min at 94 °C, 35 cycles of 30 s at 94 °C, 30 s at 47 °C, 1 min at 72 °C, and a final elongation step of 10 min at 72 °C. Three μL of each PCR product was visualized on 1.5% agarose gels stained with gel-red (Biotium). Reactions containing fragments of the expected size were purified and sequenced directly by a commercial facility (Macrogen, South Korea).

### 2.3. Sequence Analysis

**Figure 2:**
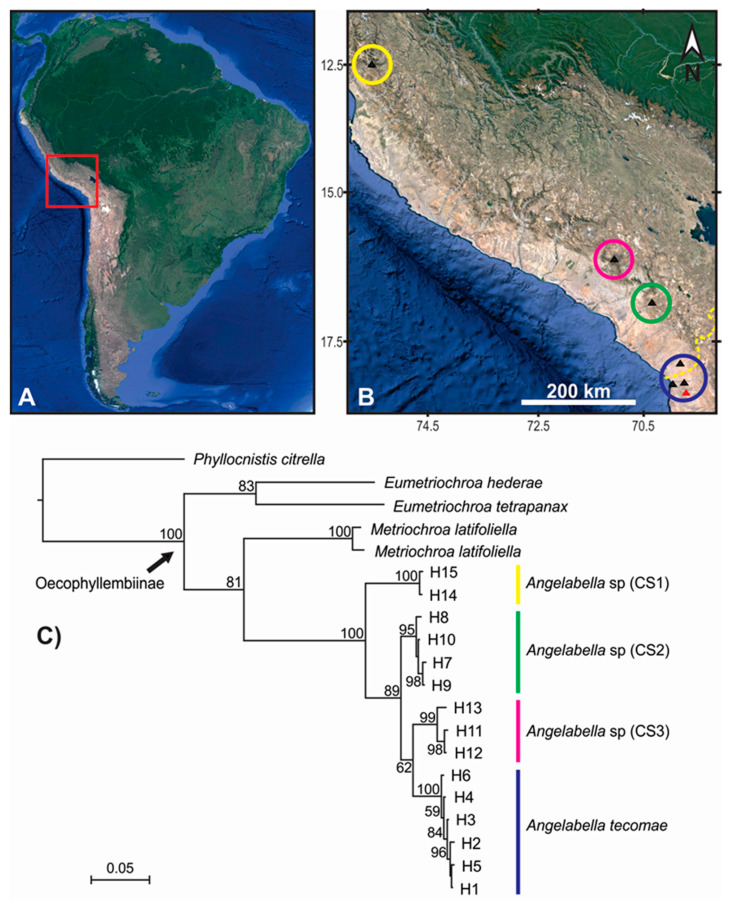
Spatial clustering and phylogenetic relationships of the COI haplotypes of *Angelabella*. (**A**) The study area (red square) in South America. (**B**) Spatial clustering of the haplotypes suggested by Geneland analysis (circles), with sampling sites marked by triangles (type locality in red). Yellow dashed line in the lower right corner of Figure 2B indicates Peru-Chile geopolitical boundary. (**C**) Phylogenetic consensus tree of *Angelabella tecomae* and candidate species (CS); color of the longitudinal stripes corresponds to that of the spatial clusters in B; numbers near branches indicate node support (posterior probability). Yellow = Lima; pink = Arequipa; green = Moquegua; blue = Tacna-Arica.

Fifteen sequences of *A. tecomae* from northern Chile (Arica) [4], including five from the type locality (Azapa Valley, red triangle in Figure 2B), were used in the analyses to evaluate the conspecificity of the 29 new Peruvian samples (Table 1). The software MEGA X [30] was used to perform the 657 bp sequence alignment with ClustalW to determine the nucleotide substitution model (under BIC criteria) and to assess the genetic distance between haplotypes with the Kimura 2-parameter (K2P) model. Genetic diversity indices were calculated in DnaSP 5.10 [31]. To assess whether the dataset represents neutral evolutionary processes, deviations from neutrality were assessed using Tajima’s D test [32] for population genetic data (*Angelabella* data only), and the Mcdonald-Kreitman (MK) test [33] for comparative data (*Angelabella* and outgroups), both analyses performed in DnaSP 5.10. The Xia test [34] was used to assess the presence of phylogenetic signal with a substitution saturation analysis in the software Dambe 7.2.1 [35].

Geneland 4.9.2 [36,37] was used to determine the spatial location of genetic discontinuities of *Angelabella*. Analyses were carried out with K = 7, with 100,000,000 MCMC iterations, thinning each 10,000 iterations and a burn-in of 10%. The number of genetic clusters was assessed through probability density distribution graphs. In parallel, the geographic barrier isolation hypothesis suggested by Geneland was evaluated by means of an analysis of molecular variance in Arlequin 3.5 [38].

To determine evolutionary relationships between haplotypes and to evaluate the reciprocal monophyly hypothesis, a phylogenetic analysis was performed by Bayesian inference using a mixture model in BayesPhylogenies [39] with a GTR+G as substitution model. To assess the monophyly of *Angelabella*, sequences of *Eumetriochroa* Kumata, *Metriochroa* Busck (Oecophyllembiinae), and *Phyllocnistis* Zeller (Phyllocnistinae) were included in the phylogenetic analysis as outgroups, following the most recent phylogeny of Gracillariidae [40]. Trees were obtained after running 200,000,000 MCMC. The parameters of the runs were observed in Tracer 1.5. The visualization of the phylogenetic tree was done in BayesTrees 1.3 [41].

To determine if the cryptic diversity suggested by Geneland and the phylogenetic analysis corresponds to different *Angelabella* species, species discovery delimitation analyses were carried out using a distance-based method, the Automatic Barcode Gap Discovery (ABGD [21]), and three tree-based methods, the single-threshold General Mixed Yule Coalescent (st-GMYC [22]), a Bayesian implementation of the General Mixed Yule Coalescent (bGMYC [42]) and a Bayesian implementation of the PTP model (bPTP [24]). The reason for using Bayesian implementations of GMYC (bGMYC) and PTP (bPTP) methods is because with the determination of evolutionary relationships under single locus approaches (usually DNA barcode sequences), the estimation of phylogenies may be associated with large amounts of uncertainty [43,44]. bGMYC incorporates uncertainty in phylogenetic relationships and allows obtaining marginal probabilities of species identities [42], and bPTP adds Bayesian support values to species delimited on the input tree, so higher Bayesian support value of a node indicates that all descendants from this node are more likely to be conspecific (https://species.h-its.org/). The ABGD analysis was performed through the web server of ABGD (http://wwwabi.snv.jussieu.fr/public/abgd/abgdweb.html) using the K2P evolution model. Outgroups were removed from alignment. A range of prior intraspecific divergence values from 0.001 to 0.3 was assayed, applying a relative gap width of 1.5. For the st-GMYC analysis, an ultrametric phylogenetic tree was generated by Bayesian inference, using the Yule process as the prior method of speciation model under a lognormal relaxed clock model in BEAST 2.5 [45] using a clock rate of 0.023 substitutions per million years [46,47]. The ultrametric tree was used as input for the st-GMYC analysis conducted in R 4.0 using the splits package (http://r-forge.r-project.org/projects/splits/). We initially included only haplotypes of *Angelabella*. However, st-GMYC results were not significantly different from the null model of coalescence. Thus we added singletons of *Metriochroa latifoliela*, *Eumetriochroa tetrapanax* and *Eumetriochroa hederae*, species from the same subfamily as *Angelabella* (Oecophillembiinae), to increase the Yule portion of the tree and fit the model to the data better (e.g., [48,49]). The bGMYC model was implemented in R 4.0 using the bGMYC package, using 100 randomly selected trees from BEAST analysis (same priors as for st-GMYC). 50,000 MCMC generations were simulated, sampling every 100 trees and discarding 10% as burn-in. Finally, to implement the bPTP method, an ML tree was determined using RAxML-NG [50] on the website https://raxml-ng.vital-it.ch/, using a GTR +G+I as the substitution model and a bootstrapping cutoff of 0.5. The best resulting RAxML tree was used as input for the bPTP analysis on the website https://species.h-its.org/, using 100,000 MCMC generations, thinning every 100 trees and discarding 10% as burn-in.

## 3. Results

No morphological differences were found between pupae of *A. tecomae* from the type locality and those collected in Lima, Arequipa, Moquegua and Tacna, neither between the genitalia of *A. tecomae* from the type locality and those of adults from Arequipa.

No evidence of stop codons or substitution saturation (ISS < ISS.C, *p* < 0.05) were detected in the alignments, indicating that datasets were suitable for all the subsequent genetic analyses.

### 3.1. Genetic Diversity and Spatial Distribution

**Figure 3:**
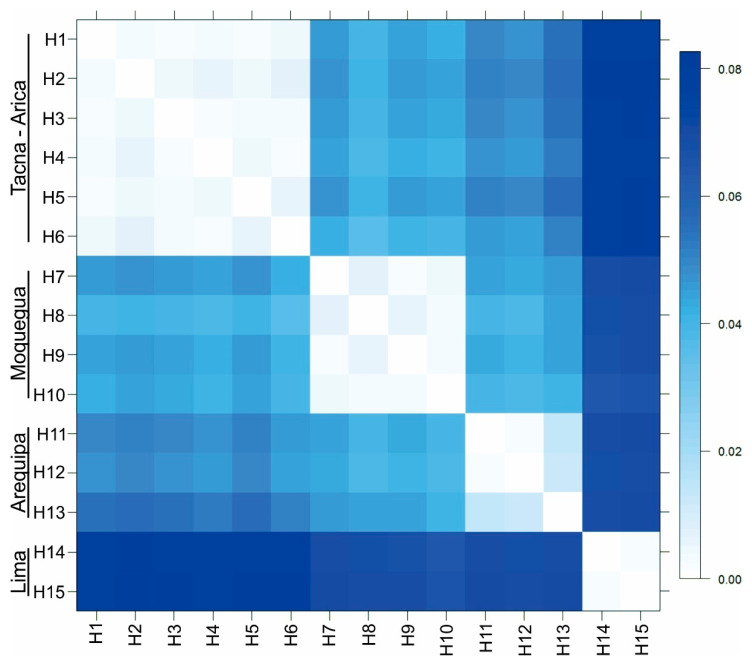
Genetic distance (K2P) matrix between COI haplotypes (657 bp) of *Angelabella* ordered according to spatial clusters suggested by Geneland. Tacna-Arica group represents *A. tecomae*, the type species of the genus.

Forty four sequences of *Angelabella* of 657 base pair (bp) length were analyzed, including 29 provided here and 15 from a previous study [4]. Their alignment showed 73 variable sites, representing fifteen haplotypes (H1-H15): two from Lima, three from Arequipa, four from Moquegua and six from Tacna-Arica. Tajima’s D neutrality test indicated a non-significant deviation from zero (Tajima’s D = 0.458, *p* > 0.1), thus supporting the null hypothesis that the observed polymorphism has been maintained without selection. The Geneland analysis indicated the maximum a posteriori estimate for four genetic clusters (K = 4) with probabilities greater than 0.4 (Figure S1). The clusters were distributed in Lima (cluster 1), Arequipa (cluster 2), Moquegua (cluster 3) and Tacna-Arica (cluster 4) (Figure 2A,B). Analysis of molecular variance indicated that 94.28% of the variation occurred between these spatial groups (F_ST_ = 0.94). Genetic distance was 0.2–0.8 and 3.6–8.3% (K2P) between haplotypes of the same and different spatial clusters, respectively (Figure 3).

### 3.2. Phylogenetic Analysis and Species Delimitation

**Figure 4:**
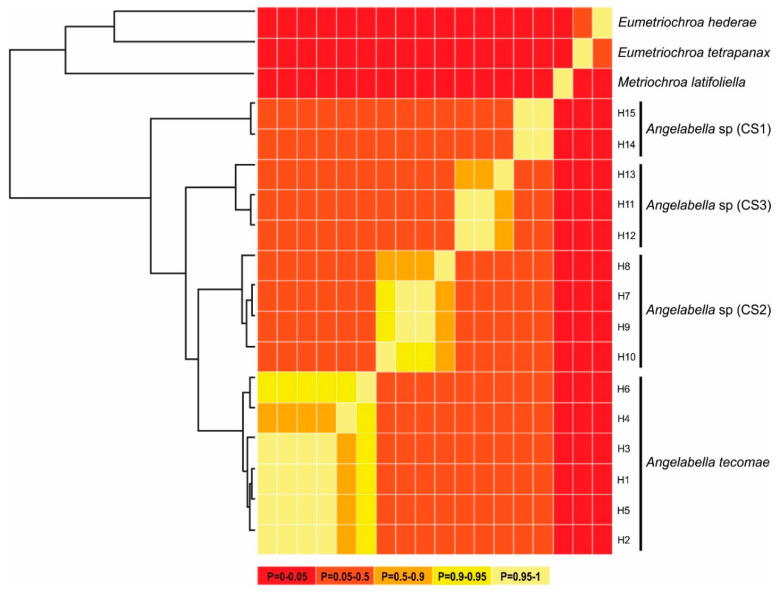
Summary of Bayesian General Mixed Yule Coalescent (bGMYC) results. Maximum clade credibility tree from BEAST (**left**) and sequence-by-sequence matrix (**right**). Colors of cells represent the posterior probability that the sequences are conspecific.

The alignment included 20 sequences of 657 bp, one of each haplotype of *Angelabella* and five of outgroups. The McDonald-Kreitman (MK) neutrality test indicated that ratio of replacement to synonymous fixed substitutions is the same as the ratio of replacement to synonymous polymorphism (G value = 0.288, *p*-value = 0.591). Therefore, the evolution represented by the dataset has occurred mainly by neutral processes. The phylogenetic analysis indicated a well-supported clade of the subfamily Oecophyllembiinae, clustering all the sequences of *Angelabella* in a clade (100% posterior probability) sister to *Metriochroa latifoliella* (Millière). The haplotypes in the *Angelabella* clade were clustered in accordance with the spatial groups suggested by Geneland in well-supported reciprocally monophyletic groups (Lima, Arequipa, Moquegua and Tacna-Arica). All species delimitation analyses (ABGD, st-GMYC, bGMYC and bPTP) indicated that *Angelabella* includes the type species, *A. tecomae*, represented by lineages of Tacna-Arica, and three candidate species (CSs) represented by the lineages of Lima, Arequipa, and Moquegua (Figure 2C). The ABGD analysis showed three barcode gaps, around 0.02, 0.04, and 0.075 (Figure S2). The st-GMYC analysis indicated that the number of *Angelabella* species delimited is four (number of ML clusters = 4; confidence interval = 2–4), favoring the st-GMYC model over the null model of coalescence (likelihood ratio = 7.501, *p*-value < 0.05), with a time threshold between inter- intraspecific branching events of about 350 Kya (Figure S3). The same number of candidate species within *Angelabella* was suggested by bGMYC analysis using a conspecificity probability threshold of 0.5 (Figure 4), assigning the same haplotypes for each candidate species as the other analyses. The highest Bayesian supported solution of the bPTP analysis indicated that *Angelabella* included four species, with ranges of support between 0.554–0.971 (Figure S4).

## 4. Discussion

The taxonomic diversity of the Neotropical Gracillariidae remains poorly researched, mainly due to the scarce collecting efforts and the small number of taxonomists focused on the study of these micromoths in this biogeographic region [13,51]. The geographic distribution is poorly documented for a large number of the currently described Neotropical gracillariids, many of which are recorded only from the type locality [52]. This is the case of the Peruvian fauna of Gracillariidae, with only 28 native species recorded despite the high plant and environmental diversity of this country [52,53,54].

It has been suggested recently that DNA barcodes can be helpful to explore geographic ranges of Lepidoptera, due to the small intraspecific divergence generally found between samples widely separated geographically [55]. In contrast, the divergence between the haplotypes of *Angelabella* of different spatial groups was relatively high on a small geographic scale. This could be attributed to the particular landscape configuration of the study area, which has relatively small patches of host plants separated by extensive hyperarid lands that appear to represent geographic barriers with low permeability to gene flow of these micromoths between patches. In relation to the geographic distribution of *A. tecomae*, the results provide molecular evidence in support of the only previous Peruvian record of this species, suggesting a relatively narrow geographic range restricted to a few ravines near the limit between Peru and Chile, as only the samples from Tacna-Arica showed divergence levels within the ranges previously reported as intraspecific for Gracillariidae [56,57,58]. This geographic range may be slightly wider, because *T. f. fulva* occurs in a few additional ravines in the southernmost part of Peru [18]; this micromoth should be searched for in all of them.

Although morphological comparisons were not an objective of this study, pupae from different sampling sites were compared under stereomicroscope before DNA extraction, and the genitalia of two males obtained from Arequipa were compared under light microscope with those of specimens from the type locality of *A. tecomae*. However, no clear differences were found in either case. The divergence between haplotypes of Angelabella from different spatial genetic clusters (3.6–8.3% K2P) is either remarkably higher than [5] or near [6,12] those recorded between morphologically cryptic species of two other genera of Gracillariidae. Similar levels of divergence have been interpreted to represent putative heterospecific lineages in the absence of morphological evidence [12,17,58]. Despite the absence of obvious morphological differentiation between samples of *Angelabella*, the deep divergence between haplotypes of different spatial clusters, their reciprocal monophyly indicated by the phylogenetic analysis and the highly consistent results of the four species delimitation analyses suggest heterospecific status for the geographically isolated lineages analyzed. Patterns of allopatric genetic differentiation similar to those found in this study have been recorded for populations of Bucculatricidae [19] and Tortricidae [59], and pairs of morphologically cryptic allopatric species are known to occur in Cosmopterigidae [60] and Tortricidae [61] near the study area, suggesting the characteristics of these hyperarid landscapes as a causal agent of allopatric diversification processes among populations of micromoths in the Peru-Chile desert.

The only other micromoth associated with *T. fulva* is the many-plumed moth *Alucita danunciae* Vargas, 2011 (Lepidoptera: Alucitidae), whose larvae feed on unripe seeds, either on *T. f. fulva* in the coastal valleys of northern Chile [62] or *T. f. arequipensis* on the western slopes of the Andes of Arequipa in southern Peru [63]. Divergence of 0.6–0.9% (K2P) was recorded between DNA barcodes of *A. danunciae* from the two localities [63], contrasting with the 4.7–5.6% (K2P) found in the present study between DNA barcodes of *Angelabella* from northern Chile and Arequipa, suggesting different lineage diversification scenarios in micromoths associated with the same host plants in the same geographic range, a comparative aspect that deserves further attention.

Although solid taxonomic identifications of Lepidoptera should be based on the analysis of a wide range of data sources, our results show that adequate single locus sequence analysis can be useful to discover surprising biodiversity patterns in underexplored environments, providing the base to plan further studies involving little-known organisms. *Angelabella* includes only the type species up to now [52]. However, the results provided here suggest that it is not a monotypic genus, but harbors four morphologically cryptic allopatric species, a scenario that should be explored further. As *Angelabella* has been surveyed only on three of the six subspecies of *T. fulva*, surveys must be expanded to the remaining three subspecies, two of which are distributed on the eastern slopes of the Andes [18], to characterize better the distribution and species diversity of this micromoth genus. A greater sampling effort is necessary to define lineage diversity at the metapopulation level for the type species and the three candidate species suggested here, which implies more detailed knowledge of their geographic ranges. Additional character sources must be assessed, including morphology of different life stages [64,65] and bi-parental markers [5,6], to understand better the processes and patterns that have governed the evolutionary history of this leaf miner genus.

## 5. Conclusions

The results obtained in this study suggest that *Angelabella* is not a monotypic genus, but harbors at least four morphologically cryptic allopatric leaf miner species with restricted geographic ranges, including the type species and three candidate species.

## Figures and Tables

**Table 1:** Sequences Used in the Analyses of this Study. Haplotypes H4 to H15 Are New.

Species	BOLD Accession	GenBank Accession	n	Locality
*Phyllocnistis citrella*	GBGL7447-11	AB614514	1	Japan
*Eumetriochroa* sp.	WOGRA322-15		1	Taiwan
*Eumetriochroa hederae*	GRPAL933-12	KF367707	1	Japan
*Metriochroa latifoliella*	GRPAL127-11	KX046527	1	France
*Metriochroa latifoliella*	GRACI588-09	HM392532	1	Croatia
*Angelabella tecomae* H1	GBGL18153-15	KM983591	4	Peru (Tacna) Chile (Arica)
*Angelabella tecomae* H2	GBGL18152-15	KM983592	9	Chile (Arica)
*Angelabella tecomae* H3	GBGL18147-15	KM983597	8	Peru (Tacna) Chile (Arica)
*Angelabella tecomae* H4		MT804725	1	Peru (Tacna)
*Angelabella tecomae* H5		MT804726	1	Peru (Tacna)
*Angelabella tecomae* H6		MT804727	2	Peru (Tacna)
*Angelabella* sp.2 H7		MT804728	2	Peru (Moquegua)
*Angelabella* sp.2 H8		MT804729	4	Peru (Moquegua)
*Angelabella* sp.2 H9		MT804730	3	Peru (Moquegua)
*Angelabella* sp.2 H10		MT804731	1	Peru (Moquegua)
*Angelabella* sp.3 H11		MT804732	1	Peru (Arequipa)
*Angelabella* sp.3 H12		MT804733	3	Peru (Arequipa)
*Angelabella* sp.3 H13		MT804734	1	Peru (Arequipa)
*Angelabella* sp.1 H14		MT804735	1	Peru (Lima)
*Angelabella* sp.1 H15		MT804736	3	Peru (Lima)

## Supplementary Materials

The following are available online at https://www.mdpi.com/2075-4450/11/10/677/s1, Figure S1: Results of Geneland analyses. A. Number of clusters along the chain after burn-in. B. Map of posterior probability to belong to clusters 1-4 (indicated by dark colors), Figure S2: Graphical results of the ABGD analysis. A. Histogram of pairwise Kimura 2-parameter distances for the COI marker, showing three barcode gaps. B. Ranked pairwise distances. C. Number of groups for partitions per value of prior intraspecific divergence, Figure S3: Results of the st-GMYC analysis. A. Number of lineages over time (years) (red vertical line indicates the threshold time between inter- intraspecific branching). B. Likelihood surface through time. C. Tree with species delimited (indicated by red branches), Figure S4: Results of the bPTP analysis. A. Highest Bayesian supported solution. B. Delimitation supports for candidate species (CS) of *Angelabella*.
