# Supplementary material for: Cryptic Diversity in the Monotypic Neotropical Micromoth Genus Angelabella (Lepidoptera: Gracillariidae) in the Peru-Chile Desert
Source: Insects. 2020 Oct 6;11(10):677. doi: 10.3390/insects11100677 (PMC7601689; doi:10.3390/insects11100677)
Supplement: Supplementary file 1 [file insects-11-00677-s001.pdf]

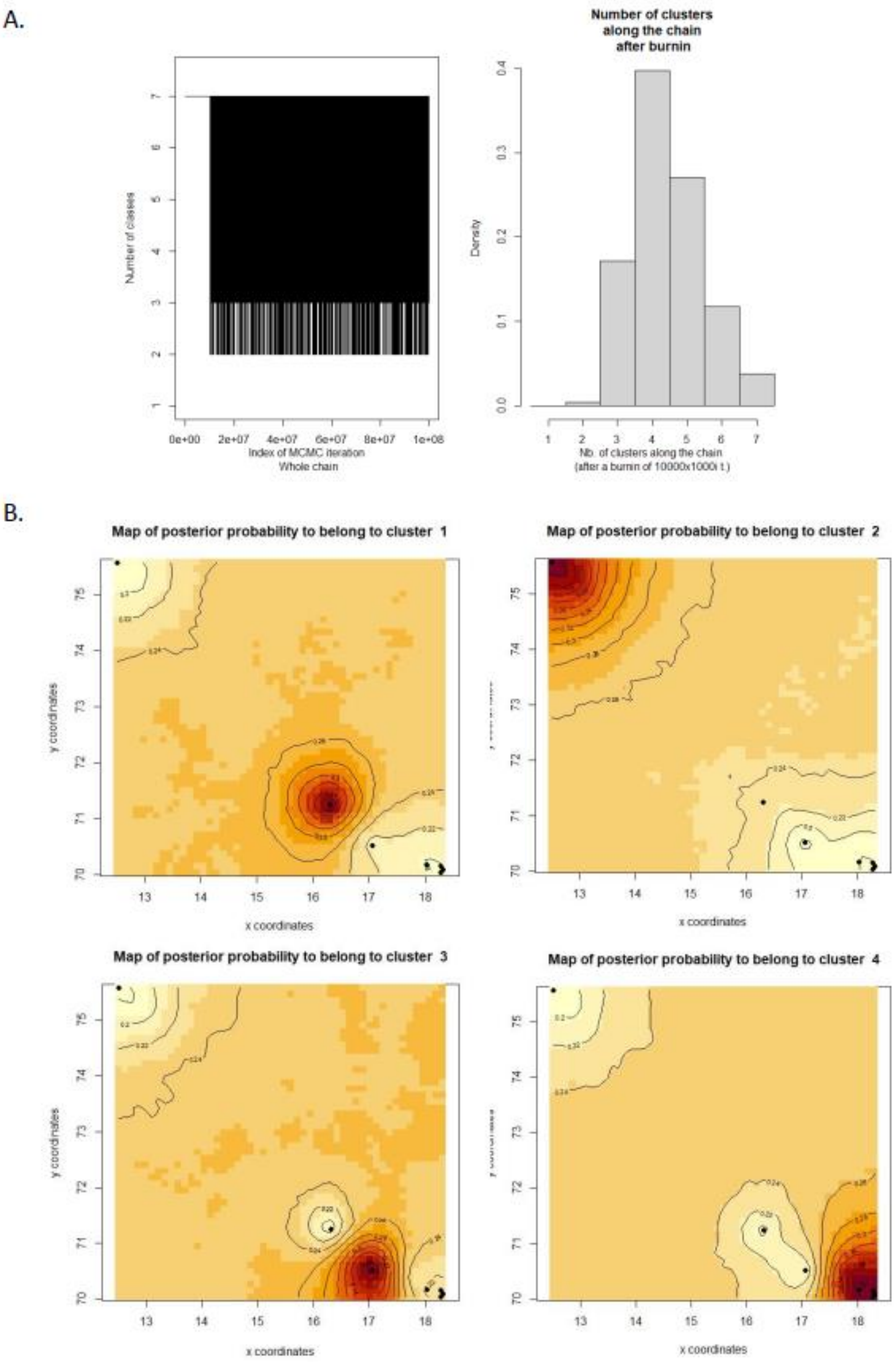

Figure S1. Results of Geneland analyses. (**A**) Number of clusters along the chain after burn-in. (**B**) Map of posterior probability to belong to clusters 1-4 (indicated by dark colors).

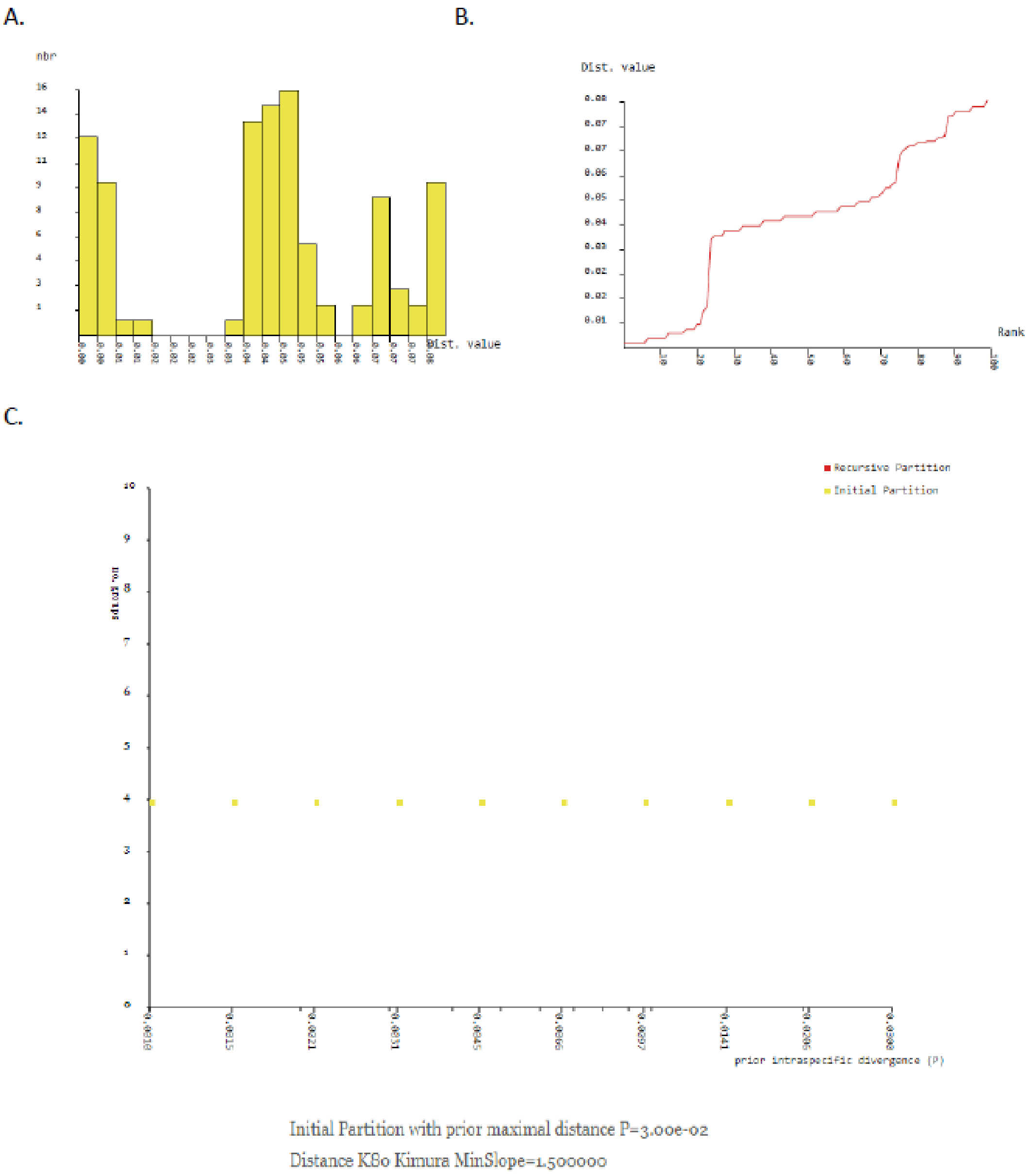

Figure S2. Graphical results of the ABGD analysis. (**A**) Histogram of pairwise Kimura 2-parameter distances for the COI marker, showing three barcode gaps. (**B**) Ranked pairwise distances. (**C**) Number of groups for partitions per value of prior intraspecific divergence.

A.

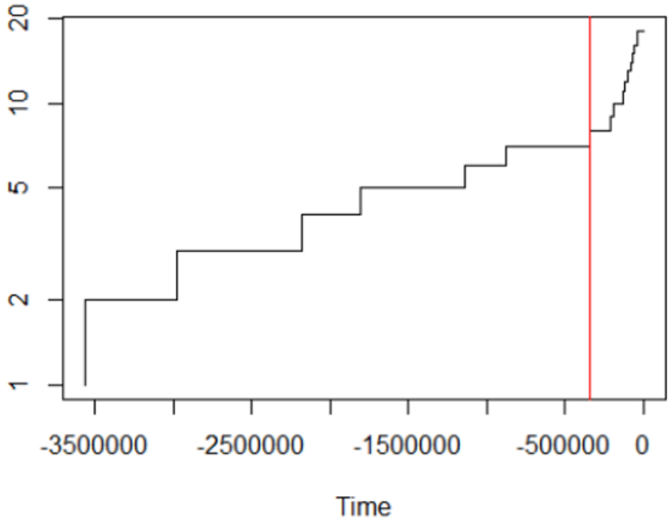

20
10
5
2
1
-3500000
-2500000
-1500000
-500000
0
Time

B.

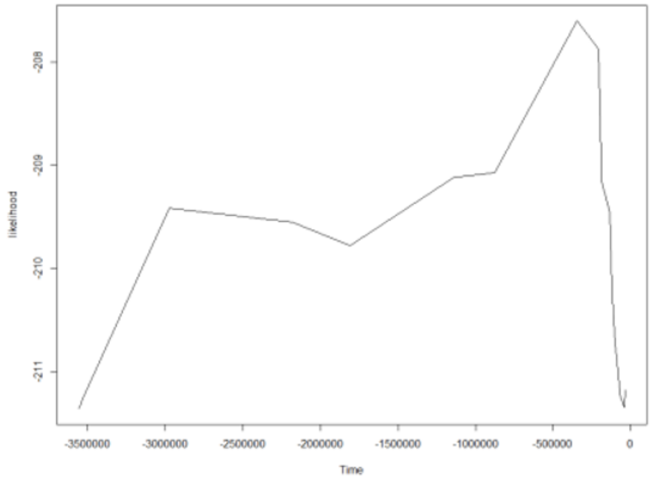

-208
-209
-210
-211
likelihood
-3500000
-3000000
-2500000
-2000000
-1500000
-1000000
-500000
0
Time

C.

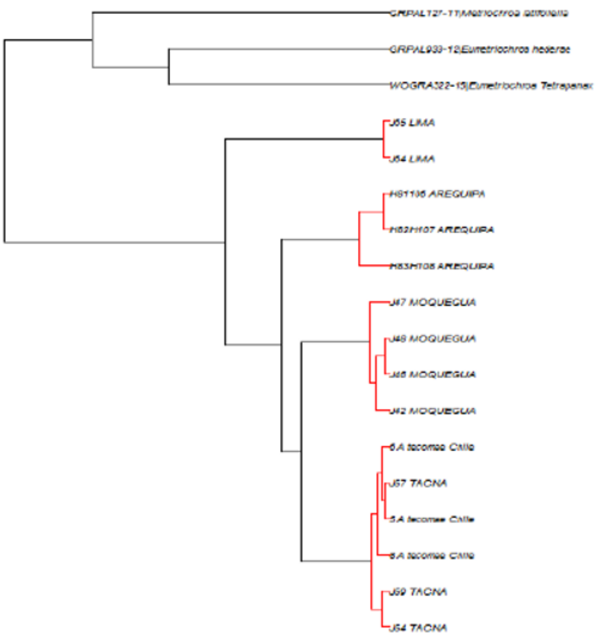

J65 LIMA
J64 LIMA
J47 MOQUEGUA
J48 MOQUEGUA
J42 MOQUEGUA
J67 TACNA
J69 TACNA
J64 TACNA

Figure S3. Results of the st-GMYC analysis. (**A**) Number of lineages over time (years) (red vertical line indicates the threshold time between inter- intraspecific branching). (**B**) Likelihood surface through time. (**C**) Tree with species delimited (indicated by red branches).

A.

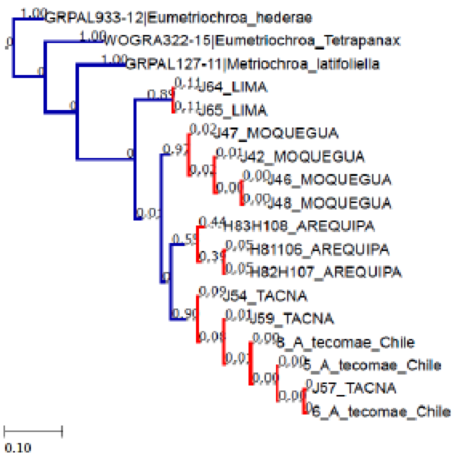

B.

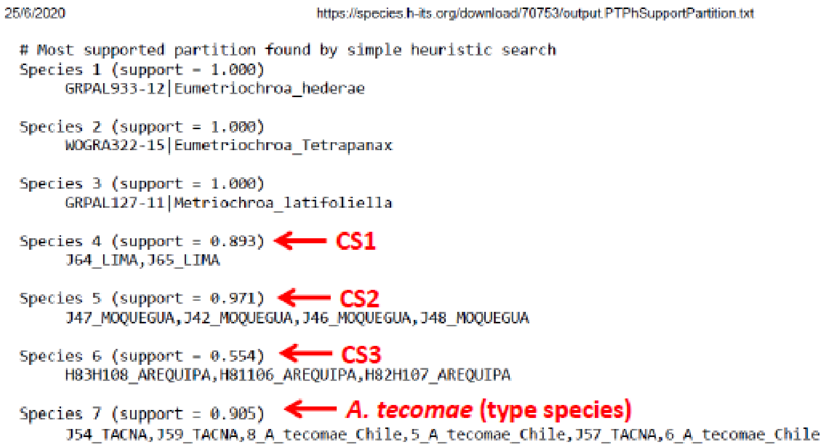
25/6/2020 https://species.h-its.org/download/70753/output.PTPhSupportPartition.txt

```
# Most supported partition found by simple heuristic search
Species 1 (support = 1.000)
      GRPAL933-12|Eumetriochroa_hederae

Species 2 (support = 1.000)
      WOGRA322-15|Eumetriochroa_Tetrapanax

Species 3 (support = 1.000)
      GRPAL127-11|Metriochroa_latifoliella

Species 4 (support = 0.893)
      J64_LIMA,J65_LIMA

Species 5 (support = 0.971)
      J47_MOQUEGUA,J42_MOQUEGUA,J46_MOQUEGUA,J48_MOQUEGUA

Species 6 (support = 0.554)
      H83H108_AREQUIPA,H81106_AREQUIPA,H82H107_AREQUIPA

Species 7 (support = 0.905)
      J54_TACNA,J59_TACNA,8_A_tecomae_Chile,5_A_tecomae_Chile,J57_TACNA,6_A_tecomae_Chile
```


Figure S4. Results of the bPTP analysis. (**A**) Highest Bayesian supported solution. (**B**) Delimitation supports for candidate species (CS) of *Angelabella*.
